# Regular AREDS2 Supplement Use in Age-Related Macular Degeneration: Factors Associated with Supplementation, Medication Adherence, and Patient-Reported Barriers

**DOI:** 10.3390/healthcare14172733

**Published:** 2026-08-27

**Authors:** Hasan Öncül, Umut Dağ, Mehmet Fuat Alakuş

**Affiliations:** Department of Ophthalmology, University of Health Sciences Diyarbakır Gazi Yaşargil Training and Research Hospital, 21070 Diyarbakır, Türkiye; dr_umut_dag@hotmail.com (U.D.); fuat2112@hotmail.com (M.F.A.)

**Keywords:** age-related macular degeneration, AREDS2, nutritional supplementation, medication adherence, physician counseling, implementation science, preventive ophthalmology

## Abstract

**Highlights:**

**What are the main findings?**

- Approximately 70% of clinically eligible patients with age-related macular degeneration (AMD) reported regular AREDS2 supplement use.
- Younger age, higher educational attainment, and physician recommendation were independently associated with regular AREDS2 supplement use.
- Among regular supplement users, only 53.5% met the study-defined criteria for good medication adherence.
- Financial burden, limited supplement availability, polypharmacy, and insufficient knowledge were the most frequently reported barriers to AREDS2 supplementation.

**What are the implications of the main findings?**

- The findings support the importance of physician counseling in the implementation of evidence-based AREDS2 recommendations in routine clinical practice.
- Improving patient education and awareness may enhance both the initiation and long-term adherence to AREDS2 supplementation.
- Reducing financial and healthcare access barriers may improve the real-world implementation of evidence-based nutritional supplementation in patients with AMD.
- Future prospective studies should evaluate interventions targeting physician counseling, patient education, and socioeconomic barriers to optimize long-term adherence and clinical outcomes.

**Abstract:**

**Background/Objectives:** Age-related macular degeneration (AMD) is a leading cause of irreversible visual impairment, and AREDS2-based nutritional supplementation is recommended for clinically eligible patients to reduce the risk of disease progression. However, successful implementation depends on regular supplement use and sustained adherence. This study investigated demographic, socioeconomic, clinical, and physician-related factors associated with regular AREDS2 supplement use and evaluated medication adherence, physician counseling, and patient-reported barriers among clinically eligible patients with AMD. **Methods:** In this single-center cross-sectional study, 389 patients with intermediate AMD or advanced AMD in one eye who fulfilled the indications for AREDS2 supplementation were enrolled. Participants reporting occasional supplement use were excluded. A structured interviewer-administered questionnaire was used to collect demographic, socioeconomic, and clinical data, together with information on physician recommendation, medication adherence, patient knowledge, and perceived barriers to supplementation. Factors associated with regular AREDS2 supplement use were evaluated using univariable and multivariable logistic regression analyses, whereas adherence patterns and patient-reported barriers were analyzed descriptively. **Results:** Of the 389 participants, 273 (70.2%) reported regular AREDS2 supplement use, whereas 116 (29.8%) were non-users. Multivariable analysis showed that younger age, educational attainment, and physician recommendation were independently associated with regular supplement use (adjusted OR for physician recommendation, 5.38; 95% CI, 2.68–10.81; *p* < 0.001). Among regular users, 53.5% met the study-defined criteria for good medication adherence. The most frequently reported barriers among non-users were financial burden, limited supplement availability, polypharmacy, and insufficient knowledge regarding AREDS2 supplementation. **Conclusions:** Regular AREDS2 supplement use among clinically eligible patients with AMD was associated with demographic, educational, and physician-related factors, whereas long-term adherence remained suboptimal despite substantial uptake. These findings emphasize the importance of effective physician counseling, structured patient education, and interventions addressing socioeconomic barriers to improve the real-world implementation of evidence-based nutritional supplementation in AMD.

## 1. Introduction

Age-related macular degeneration (AMD) is a progressive degenerative disease affecting the macula and is characterized by drusen and retinal pigmentary changes in its earlier stages, with progression to advanced atrophic or neovascular forms that may result in severe central vision loss [1,2]. AMD has a multifactorial pathogenesis involving the dysregulation of inflammatory, lipid, angiogenic, and extracellular matrix pathways [2]. It is one of the leading causes of irreversible visual impairment and blindness among older adults worldwide [1,2]. Population aging is expected to substantially increase the global burden of AMD, emphasizing the need for effective strategies to delay disease progression and preserve visual function [1]. Although intravitreal anti-vascular endothelial growth factor (anti-VEGF) therapy has transformed the management of neovascular AMD, preventive interventions remain essential because currently available treatments cannot completely halt disease progression [2]. The Age-Related Eye Disease Study (AREDS) and the subsequent AREDS2 trial demonstrated that a specific formulation of antioxidant vitamins and minerals reduces the risk of progression to advanced AMD in appropriately selected patients. Consequently, AREDS2-based nutritional supplementation is recommended for patients with intermediate AMD or advanced AMD in one eye who remain at risk of progression in the other eye [3,4,5,6].

The effectiveness of AREDS2 supplementation in routine clinical practice depends not only on its demonstrated efficacy in clinically eligible patients but also on appropriate physician recommendation, timely initiation of supplementation, and sustained long-term adherence [7,8]. Previous studies have shown that many patients have limited knowledge regarding the purpose and expected benefits of nutritional supplementation and frequently discontinue treatment because of inadequate counseling, financial burden, misconceptions regarding treatment efficacy, or concerns about adverse effects [7,8,9,10].

Previous studies have examined individual aspects of AREDS/AREDS2 implementation, including patient knowledge and adherence to recommendations [9,10,11], as well as the content and effectiveness of nutritional counseling provided by eye-care professionals [12]. Notably, Prieto Del-Cura et al. [11] evaluated adherence to AREDS2 recommendations and patient awareness among 148 patients with AMD in a Spanish tertiary-care setting and identified educational-level and other patient-related factors associated with adherence. The present study differs in both scope and analytical focus: it evaluates regular AREDS2 supplement use as the primary outcome in a larger cohort of clinically eligible patients and simultaneously examines demographic, socioeconomic, clinical, and physician-related factors associated with regular use, medication adherence among regular users, and patient-reported barriers among non-users.

Furthermore, much of the available evidence on AREDS/AREDS2 adherence, patient awareness, and supplement use has been derived from populations outside Türkiye [7,8,9,10,11,12]. Consequently, setting-specific evidence regarding the combined influence of demographic, socioeconomic, clinical, physician-related, and patient-reported factors on AREDS2 implementation in routine ophthalmic practice in Türkiye remains limited. Examining these factors in the Turkish healthcare setting is relevant because supplement availability, out-of-pocket financial burden, healthcare access, and patient education may differ across healthcare systems and populations. Thus, rather than replicating an adherence-focused assessment, the present study was designed to provide an integrated evaluation of supplement uptake, adherence after initiation, physician recommendation, and barriers to non-use within the same clinically eligible population.

Therefore, the primary aim of the present study was to identify demographic, socioeconomic, clinical, and physician-related factors associated with regular AREDS2 supplement use among clinically eligible patients with AMD. The secondary objectives were to evaluate medication adherence, physician counseling, patient knowledge, and patient-reported barriers to regular AREDS2 supplementation. We hypothesized that regular AREDS2 supplement use would be associated with demographic, socioeconomic, clinical, and physician-related factors among clinically eligible patients with AMD.

## 2. Materials and Methods

### 2.1. Study Design and Participants

This single-center, hospital-based cross-sectional study was conducted at a tertiary referral retina clinic in Türkiye. Consecutive patients with AMD who fulfilled the eligibility criteria and attended the outpatient retina clinic were recruited between March 2026 and June 2026. The study is reported in accordance with the Strengthening the Reporting of Observational Studies in Epidemiology (STROBE) guidelines for cross-sectional studies (Supplementary File S1). The study was approved by the Institutional Review Board (Protocol No. 86; Approval date: 2 March 2026) and adhered to the tenets of the Declaration of Helsinki. Written informed consent was obtained from all participants before enrollment.

The diagnosis of AMD was established by experienced retina specialists based on a comprehensive ophthalmic examination, slit-lamp biomicroscopy, dilated fundus examination, spectral-domain optical coherence tomography (SD-OCT; SPECTRALIS HRA + OCT, Heidelberg Engineering, Heidelberg, Germany), and multimodal retinal imaging when clinically indicated. Disease stage was determined according to the AREDS classification, and only patients fulfilling the established indications for AREDS2-based nutritional supplementation were included. Eligible participants included individuals with intermediate AMD in one or both eyes and those with non-neovascular advanced AMD in one eye who remained at risk of progression in the other eye.

Patients with early AMD without an indication for nutritional supplementation, those with cognitive impairment or communication difficulties preventing completion of the questionnaire, and those who declined participation were excluded.

**Figure 1:**
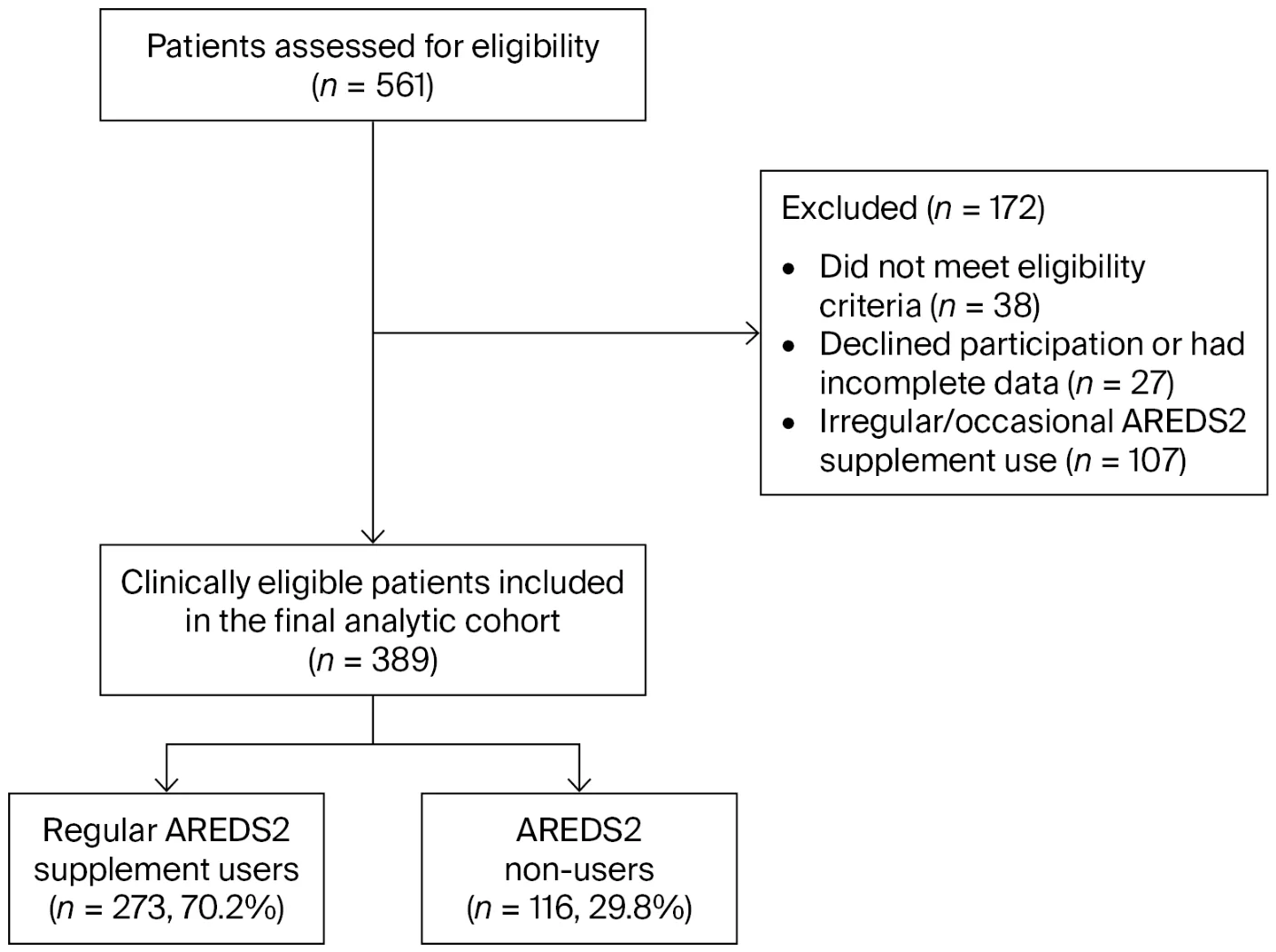
Participant flow diagram. A total of 561 patients were assessed for eligibility. Of these, 172 were excluded: 38 did not meet the eligibility criteria, 27 declined participation or had incomplete data, and 107 reported irregular/occasional AREDS2 supplement use. The final analytic cohort comprised 389 clinically eligible patients with AMD, including 273 regular AREDS2 supplement users (70.2%) and 116 non-users (29.8%).

A total of 561 patients were assessed for eligibility. Of these, 172 were excluded: 38 did not meet the eligibility criteria, 27 declined participation or had incomplete data, and 107 reported irregular or occasional AREDS2 supplement use. The final analytic cohort therefore comprised 389 clinically eligible patients, including 273 regular AREDS2 supplement users and 116 non-users (Figure 1).

Patients reporting irregular or occasional AREDS2 supplement use were excluded because the primary outcome was defined as regular supplement use, and the primary analysis was designed to compare regular users with non-users as distinct patterns of supplementation behavior. These individuals were not included in the analytic dataset, and their demographic and clinical characteristics were not systematically recorded; therefore, post hoc comparisons with regular users and non-users or multinomial sensitivity analyses could not be performed.

### 2.2. Questionnaire Development

As no validated Turkish-language questionnaire specifically addressing AREDS2 supplement use, physician counseling, patient knowledge, medication-taking behavior, and perceived barriers was available, a study-specific structured questionnaire was developed by the investigators. Item generation was informed by a review of the AREDS/AREDS2 literature, current clinical recommendations, and previous studies examining nutritional supplementation, medication adherence, patient education, and barriers to long-term treatment.

The initial item pool was independently reviewed by four retina specialists experienced in the management of age-related macular degeneration. The experts evaluated each item with regard to clinical relevance, content coverage, clarity, comprehensibility, and consistency with current AREDS2 recommendations. Based on their feedback, ambiguous wording was revised, overlapping items were removed or consolidated, and the sequence of the questions was refined. This expert-review process was undertaken to improve the content relevance and clarity of the questionnaire before its administration.

The final questionnaire included six sections assessing demographic characteristics, clinical characteristics, regular AREDS2 supplement use, medication-taking behavior, reasons for non-use, and knowledge and perceptions regarding AREDS2 supplementation. The four adherence items were combined into a predefined pragmatic adherence score developed specifically for this study for the exploratory classification of medication-taking behavior. This study-specific questionnaire was designed primarily to collect descriptive clinical information rather than to measure a single underlying psychometric construct. Therefore, formal psychometric validation, such as assessment of construct validity and test–retest reliability, was not performed. Accordingly, findings related to medication adherence should be interpreted with appropriate caution and considered exploratory. The complete study questionnaire is provided as Supplementary File S2.

### 2.3. Data Collection

All questionnaires were administered during face-to-face interviews by an ophthalmologist. Questions were read verbatim to each participant, and responses were recorded directly by the interviewer to minimize missing data and ensure consistent interpretation. Because the questionnaire was administered face-to-face by an ophthalmologist, interviewer-related and social desirability bias could not be completely excluded, particularly for self-reported supplement use and medication-taking behavior.

Demographic variables included age, sex, educational attainment, and monthly household income. Educational attainment was categorized as low (primary school or less), middle (secondary or high school), or high (university education or higher). Clinical variables included disease duration, laterality, AMD subtype, clinical stage, previous intravitreal anti-VEGF treatment, physician recommendation regarding nutritional supplementation, regular use of AREDS2-based supplements, duration of supplement use, medication adherence, and reasons for non-use. Clinical information was obtained from electronic medical records and supplemented by patient interviews.

No missing data were observed in the final analytic cohort because all included participants completed the face-to-face questionnaire.

### 2.4. Outcome Measures

The primary outcome was self-reported regular use of AREDS2-based nutritional supplementation at the time of the interview. Participants reporting regular use of AREDS2 supplements were classified as regular supplement users, whereas those reporting no supplement use were classified as non-users.

Secondary outcomes included medication adherence among regular supplement users, physician counseling, patients’ knowledge regarding AREDS2 supplementation, and self-reported barriers to supplementation among non-users.

Medication adherence among regular supplement users was assessed using a four-item structured questionnaire developed specifically for this study. Participants were asked, with reference to the past month, whether they had forgotten to take their AREDS2 supplement, whether they had stopped taking the supplement because they felt their vision was stable or they felt well, whether they had difficulty remembering to take the supplement, and whether they usually took the supplement at approximately the same time every day. For the first three items, “Yes” responses were scored as 1 and “No” responses as 0; for the fourth item, scoring was reversed (“Yes” = 0 and “No” = 1). Responses were summed to generate a total adherence score ranging from 0 to 4, with lower scores indicating better adherence. Participants with a total score of 1 or lower were classified as meeting the study-defined criteria for good adherence, whereas those with scores of 2 or higher were classified as having poor adherence. This study-specific cut-off was selected pragmatically to distinguish participants reporting no or only one adherence-related difficulty from those reporting difficulties in two or more adherence domains. The threshold was not derived from a validated adherence scale or formal psychometric analysis and should therefore be regarded as an operational definition specific to this study.

Physician counseling and patients’ knowledge regarding AREDS2 supplementation were assessed using questionnaire items specifically developed for this study. Among non-users, participants were asked to identify one or more reasons for not using AREDS2-based nutritional supplementation.

### 2.5. Statistical Analysis

Statistical analyses were performed using IBM SPSS Statistics for Windows, Version 24.0 (IBM Corp., Armonk, NY, USA). Continuous variables are presented as mean ± standard deviation (SD) or median and interquartile range (IQR), as appropriate, and categorical variables as frequencies and percentages. Comparisons between regular supplement users and non-users were performed using the independent-samples *t* test or Mann–Whitney *U* test for continuous variables and Pearson’s chi-square test or Fisher’s exact test for categorical variables, as appropriate. Factors associated with regular AREDS2 supplement use were evaluated using univariable and multivariable binary logistic regression analyses. Variables with *p* < 0.10 in univariable analysis, together with clinically relevant variables based on previous work, were included in the multivariable model. Multicollinearity was assessed using variance inflation factors (VIFs), with values < 5 considered indicative of no substantial multicollinearity, and model discrimination was assessed using the area under the receiver operating characteristic curve (AUC). The final sample included 116 participants in the smaller outcome group and 12 model parameters, corresponding to approximately 9.7 outcome events per parameter. Adjusted odds ratios (ORs) with 95% confidence intervals (CIs) were reported. Among regular supplement users, demographic and clinical characteristics were compared according to adherence status, while reasons for non-use were summarized descriptively. To account for multiple comparisons in the exploratory analyses, *p* values were adjusted using the Benjamini–Hochberg false discovery rate (FDR) procedure. Both unadjusted and FDR-adjusted *p* values are reported. All tests were two-sided, and statistical significance was defined as *p* < 0.05; for analyses subject to FDR correction, an FDR-adjusted *p* < 0.05 was considered statistically significant.

## 3. Results

A total of 389 patients with AMD were included in the study. Of these, 273 (70.2%) reported regular AREDS2 supplement use, whereas 116 (29.8%) were classified as non-users. The baseline demographic, socioeconomic, and clinical characteristics of the study population are summarized in Table 1.

Compared with non-users, regular AREDS2 supplement users were significantly younger and had higher educational attainment and monthly household income (all *p* < 0.001). They were also more likely to have bilateral disease and to report having received a physician recommendation for AREDS2 supplementation (*p* = 0.005 and *p* < 0.001, respectively). No significant differences were observed between the two groups with respect to sex, duration since AMD diagnosis, AMD subtype, previous anti-VEGF treatment, or clinical stage (Table 2).

In the univariable logistic regression analysis, younger age, higher educational attainment, higher monthly household income, bilateral disease, and physician recommendation were significantly associated with regular AREDS2 supplement use (Table 3). Among these variables, physician recommendation and high monthly household income showed the strongest unadjusted associations with regular supplement use. Sex, duration since AMD diagnosis, AMD subtype, previous anti-VEGF treatment, and clinical stage were not significantly associated with regular AREDS2 supplement use.

In the multivariable logistic regression analysis, younger age, educational attainment, and physician recommendation were identified as the key independent factors associated with regular AREDS2 supplement use (Table 4). Each one-year increase in age was associated with a 15% reduction in the odds of regular supplement use (adjusted OR, 0.85; 95% CI, 0.81–0.89; *p* < 0.001). Compared with participants with low educational attainment, those with high educational attainment had significantly greater odds of regular supplement use (adjusted OR, 4.67; 95% CI, 1.62–13.44; *p* = 0.004). In contrast, participants with middle educational attainment had significantly lower odds of regular supplement use than those with low educational attainment (adjusted OR, 0.38; 95% CI, 0.20–0.73; *p* = 0.004). Physician recommendation was associated with more than fivefold higher odds of regular AREDS2 supplement use (adjusted OR, 5.38; 95% CI, 2.68–10.81; *p* < 0.001). After adjustment for potential confounders, sex, monthly household income, duration since AMD diagnosis, clinical stage, bilateral disease, and previous anti-VEGF treatment were not independently associated with regular supplement use. The final multivariable model demonstrated good overall performance (likelihood ratio χ^2^ = 155.03, *p* < 0.001) and explained approximately 32.7% of the variability in regular AREDS2 supplement use (McFadden pseudo-*R*^2^ = 0.327). The model also demonstrated good discrimination, with an AUC of 0.864 (95% CI, 0.819–0.902). No substantial multicollinearity was detected among variables included in the multivariable model (VIF range, 1.13–3.00).

Among the 273 participants who regularly used AREDS2 supplements, 146 (53.5%) met the study-defined criteria for good medication adherence, whereas 127 (46.5%) met the study-defined criteria for poor medication adherence. Participants with good adherence were significantly younger than those with poor adherence (*p* < 0.001). Compared with participants with poor adherence, those with good adherence differed significantly with respect to sex, educational attainment, monthly household income, duration since AMD diagnosis, laterality, AMD subtype, previous anti-VEGF treatment, and clinical stage (all *p* < 0.05). No significant association was observed between adherence status and the duration of AREDS2 supplementation (*p* = 0.237) (Table 5). These univariable adherence analyses were considered exploratory because of the multiple subgroup comparisons; therefore, both unadjusted and Benjamini–Hochberg FDR-adjusted *p*-values are reported in Table 5.

Among the 116 participants who did not regularly use AREDS2 supplementation, the most frequently reported barrier was the financial cost of supplementation (67.2%). Other commonly reported barriers included limited supplement availability in pharmacies (51.7%), polypharmacy (43.1%), and insufficient knowledge regarding AREDS2 supplementation (39.7%). Approximately one quarter of participants reported difficulty accessing an ophthalmologist (27.6%) or indicated that their physician had not recommended supplementation (24.1%). Less frequently reported barriers included lack of confidence in the effectiveness of AREDS2 supplements (20.7%) and the belief that a healthy diet alone was sufficient (13.8%) (Table 6).

## 4. Discussion

The present study evaluated demographic, socioeconomic, clinical, and physician-related factors associated with regular AREDS2 supplement use among clinically eligible patients with AMD while simultaneously examining medication adherence, physician counseling, and patient-reported barriers to supplementation. Approximately 70% of eligible patients reported regular AREDS2 supplement use. However, only about one half of these patients met the study-defined criteria for good medication adherence. Multivariable analysis showed that younger age, higher educational attainment, and physician recommendation were independently associated with regular supplement use, whereas financial burden, limited supplement availability, polypharmacy, and insufficient knowledge emerged as the most frequently reported barriers among non-users. Together, these findings suggest that successful implementation of AREDS2 supplementation depends not only on clinical eligibility but also on effective physician engagement, patient education and awareness, and socioeconomic factors.

Among all variables evaluated, physician recommendation showed the strongest association with regular AREDS2 supplement use and was associated with more than fivefold higher odds of regular supplementation after adjustment for potential confounders. However, this association should be interpreted with caution because physician recommendation and supplement use are inherently interrelated in routine clinical practice, as initiation of AREDS2 supplementation would generally be expected to follow a physician’s recommendation. Therefore, the observed association should not be interpreted as evidence of an independent causal effect of physician counseling on supplement use. Rather, it may partly reflect the expected clinical pathway from recommendation to treatment initiation, as well as differences in patient–physician interaction and recall of prior counseling. Although the efficacy of AREDS2 supplementation has been firmly established by the landmark AREDS and AREDS2 randomized clinical trials [3,4,5,6], translating this evidence into routine clinical practice requires effective physician–patient communication. Our findings are consistent with those of Prieto Del-Cura et al. [11], who reported associations of ophthalmologist counseling and patient awareness with adherence to AREDS2 recommendations. Similarly, Dave et al. [12] reported substantial variability in nutritional counseling among eye-care professionals, while Giocanti-Aurégan et al. [13] emphasized the importance of physician–patient communication in promoting long-term treatment adherence in retinal diseases. Collectively, these findings underscore the importance of physician counseling as a component of AMD management and the implementation of AREDS2 recommendations in routine clinical practice.

An important strength of the present study is its comprehensive evaluation of multiple components of AREDS2 implementation within a single cohort of clinically eligible patients. Whereas previous studies have largely focused on individual aspects of supplementation, most commonly treatment adherence among existing users, our study simultaneously assessed treatment initiation, physician recommendation, medication adherence, patient knowledge, and perceived barriers. This integrated approach provides a broader understanding of the factors influencing the real-world implementation of evidence-based nutritional supplementation in AMD.

Educational attainment also remained independently associated with regular AREDS2 supplement use after adjustment for demographic, socioeconomic, and clinical characteristics. Consistent with our findings, Prieto Del-Cura et al. [11] identified educational level and patient awareness as significant determinants of adherence to AREDS2 recommendations, while Dave et al. [12] highlighted that the effectiveness of nutritional counseling depends not only on physician advice but also on patients’ ability to understand and implement the information provided. Likewise, previous studies have shown that limited knowledge of AMD is associated with poorer engagement in preventive eye care [9,10]. Together, these observations suggest that standardized educational materials and repeated counseling may improve patients’ awareness and understanding of AREDS2 supplementation and support its appropriate long-term use.

Interestingly, although higher educational attainment was associated with increased supplement use, participants with middle educational attainment demonstrated lower odds of regular supplementation than those with low educational attainment after multivariable adjustment. Because this finding has not been consistently reported in previous studies, it should be interpreted cautiously. The distribution of supplement-use status was uneven across education groups, particularly in the high-education group, in which relatively few participants were non-users. This imbalance may have contributed to the imprecision of the corresponding effect estimate, as reflected by its relatively wide confidence interval. In addition, categorizing educational attainment into three broad groups may have obscured heterogeneity within individual education levels. Therefore, the observed non-linear pattern may partly reflect category construction, sample distribution, residual confounding, or unmeasured behavioral and socioeconomic factors and requires confirmation in larger studies using a more granular assessment of educational attainment.

Monthly household income was significantly associated with regular AREDS2 supplement use in the univariable analysis but lost statistical significance after adjustment for other variables. Nevertheless, financial burden remained the most commonly reported barrier among non-users, indicating that economic factors continue to influence the real-world implementation of AREDS2 supplementation. This apparent discrepancy suggests that although income itself may not independently predict supplement use after adjustment, out-of-pocket treatment costs may still discourage patients from initiating or maintaining long-term supplementation. Similar observations have been reported previously, with treatment affordability identified as an important determinant of adherence to nutritional supplementation and other long-term ophthalmic therapies [8,12]. Furthermore, the American Academy of Ophthalmology Preferred Practice Pattern emphasizes that successful implementation of AREDS2 supplementation depends not only on appropriate physician recommendation but also on sustained patient access to recommended formulations [14].

Taken together, these findings indicate that improving the implementation of AREDS2 supplementation requires more than evidence-based prescribing alone. Interventions aimed at strengthening physician counseling, improving patient education, and reducing socioeconomic barriers may help improve the uptake of recommended nutritional supplementation among clinically eligible patients.

The present findings also demonstrate that initiating AREDS2 supplementation does not necessarily ensure sustained long-term adherence. Although all participants in this subgroup had initiated supplementation, only 53.5% met the study-defined criteria for good medication adherence. This finding is clinically relevant because the protective effect of AREDS2 supplementation depends on continuous long-term use rather than intermittent administration. As AREDS2 supplementation is preventive rather than symptom-relieving, patients may be less motivated to continue treatment when no immediate clinical benefit is perceived.

Patients with good medication adherence differed significantly from those with poor adherence with respect to age, educational attainment, monthly household income, disease duration, laterality, AMD subtype, previous anti-VEGF treatment, and disease stage. However, these adherence findings were derived from univariable comparisons and should be interpreted as exploratory. Because potential confounding was not accounted for in these analyses, the observed subgroup differences should not be interpreted as evidence that any specific patient- or disease-related characteristic was independently associated with adherence. Rather, these findings identify potential patterns that warrant confirmation in appropriately adjusted analyses in future studies.

Our findings are consistent with previous studies reporting suboptimal adherence to AREDS supplementation in routine clinical practice [7,8]. Prieto Del-Cura et al. [11] similarly identified physician counseling and patient awareness as important determinants of long-term adherence, whereas Dave et al. [12] emphasized the role of continuous lifestyle counseling in maintaining behavioral change. Collectively, these observations suggest that adherence should be considered a dynamic process requiring ongoing reinforcement throughout the follow-up rather than a single decision made at treatment initiation.

In addition to medication adherence, the present study evaluated patient-reported barriers among clinically eligible individuals who were not receiving AREDS2 supplementation. Financial burden was the most frequently reported barrier, followed by limited supplement availability, polypharmacy, and insufficient knowledge regarding AREDS2 supplementation. These findings are consistent with previous reports identifying treatment affordability and limited patient awareness as major barriers to the implementation of evidence-based nutritional recommendations [8,9,10,12]. Together with the multivariable analysis, these findings suggest that economic constraints may represent important barriers to the real-world implementation of AREDS2 supplementation.

Limited supplement availability and polypharmacy also emerged as common barriers. These factors probably reflect the practical challenges encountered by older adults managing multiple chronic diseases and complex medication regimens. Additional nutritional supplements may be perceived as burdensome or of lower priority, particularly when their benefits are preventive and become evident only over time. Future prospective studies should further investigate the influence of medication burden, healthcare accessibility, and health-system factors on long-term adherence to AREDS2 supplementation.

The present study has several strengths. It included a relatively large cohort of clinically eligible patients with AMD and simultaneously evaluated supplement use, medication adherence, physician recommendation, patient knowledge, and perceived barriers within a single study. This comprehensive approach provides a broader understanding of the real-world implementation of AREDS2 supplementation than studies focusing on a single aspect of patient behavior.

From a clinical and public health perspective, our findings suggest that successful implementation of AREDS2 supplementation requires more than recommending evidence-based nutritional supplements. A multifaceted strategy incorporating effective physician counseling, structured patient education, repeated reinforcement during follow-up, improved access to recommended formulations, and interventions addressing financial barriers may help improve both treatment initiation and long-term adherence. Such measures may support the effective implementation of evidence-based AREDS2 supplementation among clinically eligible patients.

Several limitations should be acknowledged. First, the cross-sectional design precludes causal inference; therefore, the observed associations should not be interpreted as causal relationships. Second, supplement use, medication adherence, and perceived barriers were self-reported. Furthermore, because the questionnaire was administered face-to-face by an ophthalmologist involved in the participants’ clinical care, interviewer-related and social desirability bias could not be completely excluded. Participants may have provided responses perceived as more favorable or acceptable to their treating ophthalmologist, particularly regarding supplement use, medication-taking behavior, and physician counseling. Recall bias may also have affected participants’ reporting of previous supplement use, adherence behavior, physician counseling, and reasons for non-use. These potential sources of measurement bias should be considered when interpreting the observed associations. Objective adherence measures, such as pill counts or pharmacy refill records, were not available. Third, this single-center study was conducted at a tertiary referral hospital, which may have introduced selection bias and limited the generalizability of the findings. Patients attending a tertiary retina clinic may have greater disease awareness, more frequent contact with ophthalmologists, and greater opportunities to receive treatment recommendations than the broader AMD population. Consequently, the observed rates of regular AREDS2 supplement use and medication adherence may be higher than those in community-based or general ophthalmic populations. Fourth, although the study-specific questionnaire underwent expert review to improve content relevance and clarity, it did not undergo formal psychometric validation. In addition, medication adherence was assessed using a pragmatic four-item scoring system developed specifically for this study rather than a previously validated adherence instrument. Consequently, the adherence-related findings should be interpreted with caution.

In addition, patients reporting irregular or occasional AREDS2 supplement use were excluded from the analytic cohort, and their baseline characteristics were not systematically recorded. Consequently, we were unable to compare this intermediate-use group with regular users and non-users or to perform a multinomial sensitivity analysis. Dichotomizing supplement-use status may therefore have resulted in loss of information and may have influenced the observed associations. Future prospective studies should retain irregular or occasional users as a separate category to characterize the full spectrum of supplementation behavior and assess the robustness of factors associated with different patterns of AREDS2 use.

Finally, adherence analyses were exploratory and based on univariable comparisons only; therefore, these findings require confirmation in future multicenter prospective studies using validated adherence assessment tools.

## 5. Conclusions

Among clinically eligible patients with AMD, 70.2% reported regular AREDS2 supplement use, but only 53.5% of regular users met the study-defined criteria for good medication adherence. Younger age, higher educational attainment, and physician recommendation were independently associated with regular supplement use. The observed association between physician recommendation and regular supplement use should be interpreted cautiously, as physician recommendation and treatment initiation are inherently interrelated in routine clinical practice and the cross-sectional design does not permit causal inference. Among non-users, the most frequently reported barriers were financial burden, limited supplement availability, polypharmacy, and insufficient knowledge regarding AREDS2 supplementation. These findings highlight the importance of sustained adherence, patient education, physician counseling, and improved access to recommended supplements in the real-world implementation of AREDS2 supplementation.

## Figures and Tables

**Table 1:** Baseline demographic, socioeconomic, and clinical characteristics of the study population (*N* = 389).

Variable	Overall (*N* = 389)
Age, years	68.5 ± 7.5
Age, median (IQR), years	67 (63–74)
Sex, *n* (%)	
Female	227 (58.4%)
Male	162 (41.6%)
Education level, *n* (%)	
Low	172 (44.2%)
Middle	129 (33.2%)
High	88 (22.6%)
Monthly household income, *n* (%)	
Low	48 (12.3%)
Middle	255 (65.6%)
High	86 (22.1%)
Duration since AMD diagnosis, *n* (%)	
<1 year	110 (28.3%)
1–3 years	132 (33.9%)
>3 years	147 (37.8%)
Laterality, *n* (%)	
Unilateral	211 (54.2%)
Bilateral	178 (45.8%)
AMD subtype, *n* (%)	
Non-neovascular AMD	325 (83.5%)
Neovascular AMD	64 (16.5%)
Anti-VEGF treatment, *n* (%)	
Yes	64 (16.5%)
No	325 (83.5%)
Clinical stage, *n* (%)	
Intermediate	245 (63.0%)
Advanced	144 (37.0%)
Physician recommendation, *n* (%)	
Yes	311 (79.9%)
No	78 (20.1%)

Note. Values are presented as mean ± standard deviation (SD), median (interquartile range [IQR]), or number (percentage), as appropriate. Age is presented as both mean ± SD and median (IQR). AMD, age-related macular degeneration; VEGF, vascular endothelial growth factor; IQR, interquartile range.

**Table 2:** Comparison of baseline characteristics according to regular AREDS2 supplement use.

Variable	Regular AREDS2 Supplement Users (*n* = 273)	Non-Users (*n* = 116)	*p* Value	FDR-Adjusted *p* Value
Age, years	66.3 ± 6.4	73.5 ± 7.5	<0.001	<0.003
Sex, *n* (%)			0.126	0.199
Female	152 (55.7%)	75 (64.7%)		
Male	121 (44.3%)	41 (35.3%)		
Education level, *n* (%)			<0.001	<0.003
Low	120 (44.0%)	52 (44.8%)		
Middle	73 (26.7%)	56 (48.3%)		
High	80 (29.3%)	8 (6.9%)		
Monthly household income, *n* (%)			<0.001	<0.003
Low	24 (8.8%)	24 (20.7%)		
Middle	172 (63.0%)	83 (71.6%)		
High	77 (28.2%)	9 (7.8%)		
Duration since AMD diagnosis, *n* (%)			0.185	0.231
<1 year	82 (30.0%)	28 (24.1%)		
1–3 years	85 (31.1%)	47 (40.5%)		
>3 years	106 (38.8%)	41 (35.3%)		
Laterality, *n* (%)			0.005	0.010
Unilateral	135 (49.5%)	76 (65.5%)		
Bilateral	138 (50.5%)	40 (34.5%)		
AMD subtype, *n* (%)			0.440	0.440
Non-neovascular AMD	225 (82.4%)	100 (86.2%)		
Neovascular AMD	48 (17.6%)	16 (13.8%)		
Anti-VEGF treatment, *n* (%)			0.440	0.440
Yes	48 (17.6%)	16 (13.8%)		
No	225 (82.4%)	100 (86.2%)		
Clinical stage, *n* (%)			0.139	0.199
Intermediate	165 (60.4%)	80 (69.0%)		
Advanced	108 (39.6%)	36 (31.0%)		
Physician recommendation, *n* (%)			<0.001	<0.003
Yes	242 (88.6%)	69 (59.5%)		
No	31 (11.4%)	47 (40.5%)		

Note. Data are presented as mean ± standard deviation (SD) or number (percentage), as appropriate. Continuous variables were compared using the independent-samples *t*-test, whereas categorical variables were compared using the Pearson χ^2^ test. *p*-values were adjusted for multiple comparisons using the Benjamini–Hochberg false discovery rate (FDR) procedure. An FDR-adjusted *p*-value < 0.05 was considered statistically significant. AMD, age-related macular degeneration; AREDS2, Age-Related Eye Disease Study 2; VEGF, vascular endothelial growth factor.

**Table 3:** Univariable logistic regression analysis of factors associated with regular AREDS2 supplement use.

Variable	Reference	Unadjusted OR (95% CI)	*p* Value
Age (per 1-year increase)	-	0.86 (0.83–0.90)	<0.001
Male sex	Female	1.46 (0.93–2.28)	0.101
Education level	Low		
Middle	Low	0.56 (0.35–0.91)	0.019
High	Low	4.33 (1.95–9.61)	<0.001
Monthly household income	Low		
Middle	Low	2.07 (1.11–3.87)	0.022
High	Low	8.56 (3.50–20.89)	<0.001
Duration since AMD diagnosis	<1 year		
1–3 years	<1 year	0.62 (0.35–1.08)	0.09
>3 years	<1 year	0.88 (0.50–1.55)	0.663
Bilateral disease	Unilateral	1.94 (1.24–3.05)	0.004
Neovascular AMD	Non-neovascular AMD	1.33 (0.72–2.46)	0.358
Advanced stage	Intermediate	1.45 (0.92–2.31)	0.112
Physician recommendation (Yes)	No	5.32 (3.14–9.00)	<0.001
No anti-VEGF treatment	Yes	0.75 (0.41–1.38)	0.358

Note. Odds ratios (ORs), 95% confidence intervals (CIs), and *p*-values were obtained from separate univariable logistic regression models with regular AREDS2 supplement use as the dependent variable. Continuous variables were analyzed per one-unit increase. Categorical variables were analyzed using the first category as the reference group. Statistical significance was defined as *p* < 0.05. OR, odds ratio; CI, confidence interval; AMD, age-related macular degeneration; AREDS2, Age-Related Eye Disease Study 2; anti-VEGF, anti-vascular endothelial growth factor.

**Table 4:** Multivariable logistic regression analysis of factors independently associated with regular AREDS2 supplement use.

Variable	Reference	Adjusted OR (95% CI)	*p* Value
Age (per 1-year increase)	-	0.85 (0.81–0.89)	<0.001
Male sex	Female	0.65 (0.32–1.31)	0.228
Education level	Low		
Middle	Low	0.38 (0.20–0.73)	0.004
High	Low	4.67 (1.62–13.44)	0.004
Monthly household income	Low		
Middle	Low	0.76 (0.28–2.03)	0.578
High	Low	2.56 (0.76–8.61)	0.127
Duration since AMD diagnosis	<1 year		
1–3 years	<1 year	1.01 (0.45–2.24)	0.98
>3 years	<1 year	1.42 (0.69–2.92)	0.335
Advanced clinical stage	Intermediate	1.25 (0.66–2.35)	0.5
Physician recommendation (Yes)	No	5.38 (2.68–10.81)	<0.001
Bilateral disease	Unilateral	0.68 (0.35–1.31)	0.247
No anti-VEGF treatment	Yes	1.56 (0.68–3.59)	0.293

Model performance: likelihood ratio χ^2^ = 155.03; degrees of freedom = 12; model *p* value < 0.001; McFadden pseudo *R*^2^ = 0.327; model convergence = successful. Note. Adjusted odds ratios (ORs) and 95% confidence intervals (CIs) were estimated using a multivariable logistic regression model with regular AREDS2 supplement use as the dependent variable. Variables entered into the model included age, sex, education level, monthly household income, duration since AMD diagnosis, clinical stage, physician recommendation for AREDS2 supplementation, laterality, and anti-VEGF treatment. Variables with multiple categories were analyzed using indicator (dummy) variables, with the first category serving as the reference. Statistical significance was defined as *p* < 0.05. OR, odds ratio; CI, confidence interval; AMD, age-related macular degeneration; AREDS2, Age-Related Eye Disease Study 2; VEGF, vascular endothelial growth factor.

**Table 5:** Comparison of patients according to medication adherence among regular AREDS2 supplement users (*n* = 273).

Variable	Good Adherence (*n* = 146)	Poor Adherence (*n* = 127)	*p* Value	FDR-Adjusted *p* Value
Age, years	64.4 ± 5.9	68.6 ± 6.1	<0.001	<0.002
Sex, *n* (%)			0.032	0.040
Female	72 (49.3%)	80 (63.0%)		
Male	74 (50.7%)	47 (37.0%)		
Education level, *n* (%)			<0.001	<0.002
Low	84 (57.5%)	36 (28.3%)		
Middle	30 (20.5%)	43 (33.9%)		
High	32 (21.9%)	48 (37.8%)		
Monthly household income, *n* (%)			<0.001	<0.002
Low	4 (2.7%)	20 (15.7%)		
Middle	110 (75.3%)	62 (48.8%)		
High	32 (21.9%)	45 (35.4%)		
Duration since AMD diagnosis, *n* (%)			<0.001	<0.002
<1 year	43 (29.5%)	39 (30.7%)		
1–3 years	61 (41.8%)	24 (18.9%)		
>3 years	42 (28.8%)	64 (50.4%)		
Laterality, *n* (%)			<0.001	<0.002
Unilateral	36 (24.7%)	99 (78.0%)		
Bilateral	110 (75.3%)	28 (22.0%)		
AMD subtype, *n* (%)			0.002	0.003
Non-neovascular AMD	110 (75.3%)	115 (90.6%)		
Neovascular AMD	36 (24.7%)	12 (9.4%)		
Anti-VEGF treatment, *n* (%)			0.002	0.003
Yes	36 (24.7%)	12 (9.4%)		
No	110 (75.3%)	115 (90.6%)		
Clinical stage, *n* (%)			0.016	0.023
Intermediate	78 (53.4%)	87 (68.5%)		
Advanced	68 (46.6%)	40 (31.5%)		
Duration of AREDS2 supplementation, *n* (%)			0.237	0.237
<6 months	8 (5.5%)	4 (3.1%)		
6–12 months	57 (39.0%)	51 (40.2%)		
1–3 years	33 (22.6%)	40 (31.5%)		
>3 years	48 (32.9%)	32 (25.2%)		

Note. Values are presented as mean ± standard deviation (SD) or number (percentage), as appropriate. Comparisons between patients with good and poor adherence were performed using the independent-samples *t*-test for continuous variables and the Pearson χ^2^ test for categorical variables. Good adherence was defined according to the predefined adherence scoring system. *p*-values were adjusted for multiple comparisons using the Benjamini–Hochberg false discovery rate (FDR) procedure. An FDR-adjusted *p*-value < 0.05 was considered statistically significant. AMD, age-related macular degeneration; AREDS2, Age-Related Eye Disease Study 2; VEGF, vascular endothelial growth factor.

**Table 6:** Reported reasons for non-use of AREDS2 supplementation among non-users (*n* = 116).

Reason for Non-Use	*n*	%
High cost of supplementation	78	67.2
Unable to obtain the supplement from the pharmacy	60	51.7
Polypharmacy (already taking multiple medications)	50	43.1
Lack of knowledge about AREDS2 supplements	46	39.7
Difficulty accessing an ophthalmologist	32	27.6
Physician did not recommend supplementation	28	24.1
Do not believe the supplements are effective	24	20.7
Belief that natural nutrition is sufficient	16	13.8

Note. Participants were allowed to select more than one reason; therefore, percentages do not sum to 100%. Percentages were calculated using the number of participants who did not regularly use AREDS2 supplementation (*n* = 116) as the denominator. AREDS2, Age-Related Eye Disease Study 2.

## Data Availability

The datasets generated and/or analyzed during the current study are available from the corresponding author on reasonable request due to ethical and data protection restrictions related to research involving human participants.

## Supplementary Materials

The following supporting information can be downloaded at: https://www.mdpi.com/article/10.3390/healthcare14172733/s1, Supplementary File S1: STROBE Statement—Checklist of Items That Should Be Included in Reports of Cross-Sectional Studies; Supplementary File S2: Study Questionnaire.

## Abbreviations

The following abbreviations are used in this manuscript:

AMD	Age-related macular degeneration
AREDS	Age-Related Eye Disease Study
AREDS2	Age-Related Eye Disease Study 2
SD-OCT	Spectral-domain optical coherence tomography
anti-VEGF	Anti-vascular endothelial growth factor
