# Supplementary material for: Regular AREDS2 Supplement Use in Age-Related Macular Degeneration: Factors Associated with Supplementation, Medication Adherence, and Patient-Reported Barriers
Source: Healthcare (Basel). 2026 Aug 27;14(17):2733. doi: 10.3390/healthcare14172733 (PMC13564648; doi:10.3390/healthcare14172733)
Supplement: Supplementary file 1 [file healthcare-14-02733-s001.zip › Supplementary File S2.pdf]

Supplementary File S2

Study Questionnaire

Analysis of Determinants and Barriers to AREDS2 Supplement Use in Patients with Age-Related Macular Degeneration: A Single-Center Cross-Sectional Study

---

Section 1. Demographic Characteristics

1. Age: __________ years

2. Sex

☐ Female

☐ Male

3. Educational attainment

☐ Low

☐ Middle

☐ High

4. Monthly household income

☐ Low

☐ Middle

☐ High

Section 2. Clinical Characteristics

5. Duration since AMD diagnosis

☐ <1 year

☐ 1–3 years

☐ >3 years

6. Disease laterality

☐ Unilateral

☐ Bilateral

7. AMD subtype

☐ Non-neovascular AMD

☐ Neovascular AMD

8. Previous intravitreal anti-VEGF treatment

☐ Yes

☐ No

9. Clinical stage

☐ Intermediate AMD

☐ Advanced AMD

10. Has your ophthalmologist recommended AREDS2 supplementation during your most recent visit?

☐ Yes

☐ No

Section 3. AREDS2 Supplement Use (Primary Outcome)

11. Do you currently use AREDS2-based nutritional supplements for your eye disease?

☐ I use them regularly.

☐ I do not use them.

If "I do not use them" is selected, please skip to Section 5.

12. Duration of AREDS2 supplement use

☐ <6 months

☐ 6–12 months

☐ 1–3 years

☐ >3 years

Section 4. Medication Adherence

During the past month:

13. Have you ever forgotten to take your AREDS2 supplement?

☐ Yes

☐ No

14. Have you ever stopped taking your supplement because you felt your vision was stable or you felt well?

☐ Yes

☐ No

15. Do you have difficulty remembering to take your supplement?

☐ Yes

☐ No

16. Do you usually take your supplement at approximately the same time every day?

☐ Yes

☐ No

Researcher Scoring

Questions 13–15

Yes = 1

No = 0

Question 16

Yes = 0

No = 1

Total adherence score: **0–4**

Good adherence: 0–1

Poor adherence: 2–4

Section 5. Reasons for Not Using AREDS2 Supplements

17. If you do not use AREDS2 supplements, what are the reasons? (More than one response may be selected.)

☐ My ophthalmologist did not recommend them.

☐ I do not believe they are effective.

☐ I am concerned about possible side effects.

☐ The cost is too high.

☐ I already take too many medications.

☐ I cannot obtain them from the pharmacy.

☐ I believe a healthy diet alone is sufficient.

☐ I have difficulty accessing an ophthalmologist.

☐ I do not have enough information about AREDS2 supplements.

Section 6. Knowledge and Perceptions Regarding AREDS2 Supplementation

Please indicate your level of agreement with each statement.

1 = Strongly disagree

2 = Disagree

3 = Neutral

4 = Agree

5 = Strongly agree

18. AREDS2 supplementation helps reduce the risk of progression of age-related macular degeneration.

1 ☐ 2 ☐ 3 ☐ 4 ☐ 5 ☐

19. My ophthalmologist provided sufficient information about AREDS2 supplementation.

1 ☐ 2 ☐ 3 ☐ 4 ☐ 5 ☐

20. The cost of AREDS2 supplements represents a financial burden for me.

1 ☐ 2 ☐ 3 ☐ 4 ☐ 5 ☐

21. I would like to receive more information about AREDS2 supplementation.

1 ☐ 2 ☐ 3 ☐ 4 ☐ 5 ☐

22. I am concerned about the possible side effects of AREDS2 supplements.

1 ☐ 2 ☐ 3 ☐ 4 ☐ 5 ☐
