# Supplementary material for: Regular AREDS2 Supplement Use in Age-Related Macular Degeneration: Factors Associated with Supplementation, Medication Adherence, and Patient-Reported Barriers
Source: Healthcare (Basel). 2026 Aug 27;14(17):2733. doi: 10.3390/healthcare14172733 (PMC13564648; doi:10.3390/healthcare14172733)
Supplement: Supplementary file 1 [file healthcare-14-02733-s001.zip › Supplementary File S1.pdf]

Supplementary File S1

STROBE Statement—Checklist of Items That Should Be Included in Reports of Cross-Sectional Studies

Section/Item	Recommendation	Reported on Page No.
**Title and Abstract**		
1(a)	Indicate the study's design with a commonly used term in the title or abstract	Title; Abstract
1(b)	Provide an informative and balanced summary	Abstract
**Introduction**		
2	Explain the scientific background and rationale	Introduction
3	State specific objectives and prespecified hypotheses	Introduction
**Methods**		
4	Present key elements of study design	Materials and Methods – Study Design and Participants
5	Describe the setting, locations, and relevant dates	Study Design and Participants
6(a)	Describe eligibility criteria and participant selection	Study Design and Participants
7	Clearly define outcomes, exposures, predictors, and confounders	Outcome Measures
8	Describe data sources and methods of assessment	Questionnaire Development; Data Collection
9	Describe efforts to address potential sources of bias	Data Collection; Limitations
10	Explain how study size was determined	Study Design and Participants
11	Explain handling of quantitative variables	Statistical Analysis
12(a)	Describe all statistical methods	Statistical Analysis
12(b)	Describe methods for subgroup analyses	Statistical Analysis
12(c)	Explain how missing data were addressed	Data Collection; Statistical Analysis
**Results**		
13(a)	Report participant numbers at each stage	Results
13(b)	Give reasons for non-participation	Results; Study Design and Participants
13(c)	Consider use of a flow diagram	Not applicable
14(a)	Describe participant characteristics	Table 1; Results
14(b)	Indicate missing data for each variable	No missing data

Section/Item	Recommendation	Reported on Page No.
15	Report outcome events or summary measures	Results; Tables 2–6
16(a)	Present unadjusted and adjusted estimates with precision	Tables 3 and 4
16(b)	Report category boundaries where applicable	Tables
16(c)	Translate estimates into clinically meaningful terms if relevant	Results
17	Report other analyses performed	Results
**Discussion**		
18	Summarize key results	Discussion
19	Discuss study limitations	Discussion
20	Provide overall interpretation	Discussion
21	Discuss generalizability	Discussion
**Other Information**		
22	Report funding source and role of funders	Funding Statement
